# Under-five mortality from unintentional suffocation in China, 2006-2016

**DOI:** 10.7189/jogh.09-010602

**Published:** 2019-06

**Authors:** Lijun Wang, Yuyan Gao, Peng Yin, Peixia Cheng, Yunning Liu, David C Schwebel, Jiangmei Liu, Jinlei Qi, Maigeng Zhou, Guoqing Hu

**Affiliations:** 1 National Center for Chronic and Non-communicable Disease Control and Prevention, Chinese Center for Disease Control and Prevention, Beijing, China; 2 Department of Epidemiology and Health Statistics, Xiangya School of Public Health, Central South University, Changsha, China; 3 Department of Psychology, University of Alabama at Birmingham, Birmingham, Alabama, USA; * Joint first authors in this position

## Abstract

**Background:**

We used nationally representative data to examine trends in under-five unintentional suffocation mortality from 2006 to 2016 in China and mortality differences across age groups, sexes, rural vs urban locations and injury mechanisms.

**Methods:**

Mortality data came from 161 surveillance points of China’s disease surveillance points (DSPs) system. Unintentional suffocation deaths were identified through the 10th International Classification of Disease (ICD-10 codes: w75-w84). Negative binomial regression tested the significance of change in overall and subgroup mortality between 2006 and 2016.

**Results:**

Despite minor fluctuations, a steady trend in overall age-adjusted unintentional suffocation mortality was observed from 2006 to 2016 in Chinese children under 5 years of age. Infants (<1 year), boys and rural children had higher mortality rates than children aged 1-4 years, girls and urban children, respectively. Strangulation and suffocation in bed was the most common cause of mortality for infants, accounting for 66% of deaths. Children aged 1-4 years suffered more often from inhalation suffocation (55% of deaths).

**Conclusions:**

Unintentional suffocation mortality rates in under-five children remained fairly stable in China over the past decade but remained at high levels. We discuss actions that might be implemented to reduce pediatric suffocation rates in China.

Unintentional suffocation is a major cause of death for children under five years in China, causing about 3231 deaths in 2016 [1]. Mortality among young children in China (5.33 per 100 000 persons) is far higher than other nations, including the US (3.31 per 100 000 persons), Canada (1.2 per 100 000 persons), and Australia (0.8 per 100 000 persons), and suffocation deaths among young children in China account for an estimated 28% of unintentional suffocation deaths to children in that age group globally [1]. Previous studies in China suggest unintentional suffocation was the leading cause of injury deaths for infants (<1 year old) in multiple areas of the country, including Hunan province [2], Henan province [3,] Beijing city [4], and Guangzhou city [5] of China, and that children in rural areas had higher unintentional suffocation incidence and mortality rates than those in urban areas [2,4-8].

The only published national study examining under-five unintentional suffocation mortality used data from the Chinese Maternal and Child Mortality Surveillance (MCMS) system; it reported a substantial mortality decrease between 2000 and 2010, plus higher unintentional suffocation mortality in rural areas in both 2000 and 2010 compared to urban areas [6]. Several other published studies investigated the epidemiology of under-five unintentional suffocation mortality for Hunan province [2], Beijing city [4,9], Guangzhou city [5] and Sichuan province [7], revealing a declining trend in mortality over time and higher mortality rates in boys and rural children than in girls and urban children.

To date, no published studies from China examine sex-, age-, and mechanism-specific unintentional suffocation mortality and change over time on a nationwide scale. The best available data, from the Global Burden of Disease (GBD) estimates [1], include information on sex- and age-based trends over time, but no estimates of unintentional suffocation mortality by mechanism. Therefore, using data from China’s disease surveillance points (DSPs) system, we conducted a longitudinal analysis to examine changes in under-five unintentional suffocation mortality from 2006 to 2016, including mortality differences across sex, age group, location (urban/rural) and mechanism of suffocation.

## METHODS

Mortality data were retrieved from the Disease Surveillance Points (DSPs) data set, a nationally representative source of health information in China [10]. The DSPs system includes urban and rural surveillance points (districts and counties) that are randomly selected from throughout the country, and all residents within each surveillance point are covered by the DSPs. The causes of death are determined by trained coders from local hospitals and the Chinese national Center for Disease Control and Prevention (CDC) based on the 10th Edition of the International Classification of Diseases (ICD-10) and are reported to the superior CDC according to a standardized protocol [11]. A routine, internal procedural check system is used to identify and logic report errors by the DSPs, such as duplicate reports [11]. In addition, a fixed sample survey is conducted at all surveillance points every three years to adjust for underreporting [12]. In 2008, a web-based online reporting system was introduced to improve the timeliness of data reporting of the DSPs [9].

**Figure 1:**
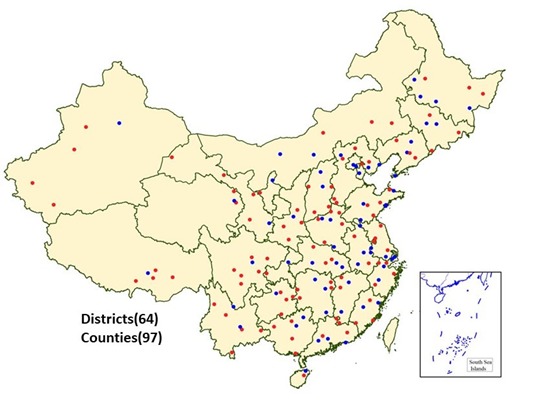
Geographical distribution of 161 surveillance points of Disease Surveillance Points (DSP) of China.

The number of surveillance points used in the DSPs was expanded in 2004-2006 and again in 2013 [13,14]. To control for the potential impact of DSPs expansions [14], we limited our analysis to data from the 161 surveillance points (64 urban points and 97 rural points) (See **Figure 1** for map of included locations) that had continuous surveillance data from 2006 to 2016.

Sex, location (urban/rural), age group and mechanism of suffocation were considered in data analysis. Under-five mortality rates from 2006-2016 were calculated as number of number of deaths divided by the corresponding population and then were age-adjusted using the age structure from the 2010 Chinese national census (<1 year and 1-4 years). In addition to reporting under-five mortality, we conducted analyses for the two age groups separately because developmental factors lead to great mortality gaps in suffocation rates between children under age 1 vs 1-4 years old [2,4,5,15] Following the ICD-10, we limited unintentional suffocation to ICD-10 codes “w75-w84”. We further divided unintentional suffocation into four groups according to the mechanism: (1) suffocation and strangulation in bed (w75); (2) inhalation-related suffocation (w78, w79, w80); (3) other specified suffocations (w76, w77, w81, w83); and (4) suffocations with unspecified cause (w84).

Percent change in mortality between 2006 and 2016 was estimated based on mortality rate ratio, which was calculated as (mortality rate ratio – 1)×100% (note: mortality rate ratio is denoted as “*eb*” and obtained via negative binomial regression; *e* approximately equals 2.7183 and *b* signifies the regression coefficient). Using standard errors of regression coefficients, we further calculated 95% confidence intervals (95% CI) of mortality rate ratio and percent change in mortality between 2006 and 2016. All statistical analyses were conducted using Stata 12.1. *P<*0.05 was considered statistically significant.

## RESULTS

From 2006 to 2016, the 161 surveillance points of DSPs reported 2937 under-five child deaths from unintentional suffocation. (**Table 1**) Under-five unintentional suffocation mortality rates showed a general increasing trend from 2006 to 2013 (with some fluctuation between 2007 and 2009), rising from 4.40 to 6.45 per 100 000 population. Between 2013 and 2016, mortality rates continuously decreased to 4.45 per 100 000 population in 2016.

**Table 1:** Distribution unintentional suffocation deaths for under-5 children in 161 surveillance points of DSP of China, 2006-2016

Age group	Sex/location	Number of deaths										
2006	2007	2008	2009	2010	2011	2012	2013	2014	2015	2016
<1 year	Total	182	192	276	233	241	236	186	216	185	163	150
	Sex:											
	boy	100	113	154	122	154	141	107	122	106	94	94
	girl	82	79	122	111	87	95	79	94	79	69	56
	Location:											
	urban	42	42	60	53	52	52	34	42	44	51	45
	rural	140	150	216	180	189	174	152	174	141	112	105
1-4 years	Total	40	52	69	63	60	60	61	78	72	58	64
	Sex:											
	boy	26	34	45	39	42	35	41	48	54	36	46
	girl	14	18	24	24	18	25	20	30	18	22	18
	Location:											
	urban	8	13	9	7	14	5	20	19	24	15	12
	rural	32	39	60	56	46	55	41	59	48	43	52

**Figure 2:**
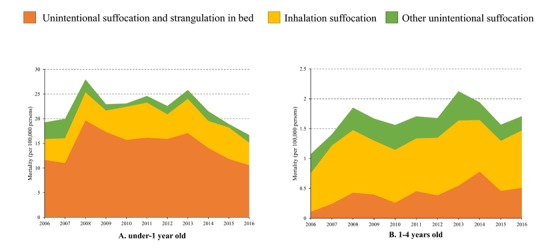
Unintentional suffocation mortality among under-5 children by mechanism in China, 2006-2016.

For under-one children, unintentional suffocation mortality fluctuated greatly from 2006 to 2016, with one peak in 2008 (27.98 per 100 000 population) and another peak in 2013 (25.84 per 100 000 population). The under-one unintentional suffocation mortality rose by 34% between 2006 and 2013 and then decreased by 35% between 2013 and 2016 (**Table 2**). Boys and rural children under one year had higher unintentional suffocation mortality rates than girls and urban children across the study time period. All subgroups followed a similar changing pattern in unintentional suffocation mortality rate from 2006 to 2016. Panel A in **Figure 2** shows that unintentional suffocation and strangulation in bed was the most common mechanism of suffocations for under-one children, accounting for 66% of fatal unintentional suffocations among that age group between 2006 and 2016. Remarkably, bed-related suffocation mortality rates decreased 9% between 2006 and 2016, from 11.65 to 10.57 per 100 000 population.

**Table 2:** Change in unintentional suffocation mortality for under-5 children in China, 2006-2016

Age group	Sex/location	Mortality rate (per 100 000 persons)											Percent change in rate (%, 95% CI)*	Pseudo R-squared
2006	2007	2008	2009	2010	2011	2012	2013	2014	2015	2016
<1 year	Total	19.28	20.00	27.98	22.94	23.10	24.62	22.59	25.84	21.52	18.94	16.70	-13 (-70, 8)	0.25
	Sex:													
	boy	20.81	22.76	30.19	23.22	28.54	28.34	23.98	26.84	22.70	20.16	19.28	-7 (-70, 23)	0.22
	girl	17.69	17.04	25.61	22.63	17.27	20.61	20.95	24.64	20.11	17.49	13.63	-23 (-55, 8)	0.24
	Location:													
	urban	13.56	13.47	19.06	16.93	16.88	22.61	12.85	15.94	15.74	18.19	14.85	10 (-72, 67)	0.18
	rural	22.07	23.13	32.16	25.61	25.70	25.43	27.21	30.39	24.30	19.30	17.63	-20 (-62, 3)	0.26
1-4 years	Total	1.07	1.40	1.85	1.67	1.56	1.71	1.68	2.13	1.94	1.57	1.71	60^†^ (7, 136)	0.20
	Sex:													
	boy	1.36	1.77	2.32	1.99	2.10	1.91	2.08	2.41	2.67	1.79	2.26	66† (2, 168)	0.17
	girl	0.77	1.01	1.34	1.32	0.98	1.49	1.20	1.80	1.07	1.30	1.06	38 (-68, 176)	0.17
	Location:													
	urban	0.66	1.07	0.73	0.56	1.13	0.45	1.66	1.57	1.88	1.16	0.89	35 (-56, 233)	0.30
	rural	1.28	1.57	2.41	2.21	1.77	2.28	1.68	2.40	1.97	1.78	2.17	70 (9, 164)†	0.21

CI – confidence interval

*Percent change in rate was calculated as (mortality in 2016 – mortality in 2006) / mortality in 2006 × 100%.

†*P* < 0.05.

During the same period, unintentional suffocation mortality increased 60% among children aged 1-4 years, rising from 1.07 to 1.71 per 100 000 population (**Table 2**). The mortality rate did not change significantly over time for girls and urban children, but increased substantially for boys (66%, 95% CI = 2%-168%) and rural children (70%, 95% CI = 9%-164%) between 2006 and 2016 (**Table 2**). Boys and rural children were at higher risk of fatal suffocation compared to girls and urban children across all years among 1- to 4-year olds. As shown in panel B in **Figure 2**, inhalation suffocation was the leading mechanism of suffocation for children aged 1-4 years, causing 55% of unintentional suffocation deaths to that age group between 2006 and 2016. Inhalation suffocation mortality increased from 0.64 to 0.96 per 100 000 persons between 2006 and 2016 for children aged 1-4 years.

## DISCUSSION

Our analysis of unintentional suffocation among Chinese children ages 0-4 between 2006 and 2016 leads to three major findings: (1) unintentional suffocation mortality rates increased substantially between 2006 and 2016 among Chinese children aged 1-4 years, with the increase occurring primarily for boys and rural children; (2) children living in rural areas and boys had higher unintentional suffocation mortality rates than children in urban areas and girls, and this finding crossed all children ages 0-4 but was particularly notable for children in the 1-4 year group; and (3) the major mechanisms for fatal unintentional suffocation were strangulation and suffocation in bed for under-one children, and inhalation suffocation for children ages 1-4 years.

The change patterns revealed by this study in China differ from the upward trends reported for the US from 1999 to 2015 [16], and the declining trends reported for Japan (2000-2009) and South Korea (1996-2006) over similar time periods [17,18]. The trend also differs from the recent decline reported by previous studies in single Chinese provinces and cities based on the MCMS data, including reports from Hunan province (2009-2014) [2], Guangzhou city (2001-2010) [5]. Sichuan province (2001-2013) [7], and Beijing city (1992-2015) [9]. Our estimates are comparable to GBD estimates, the only nationally-available published data [1].

Inconsistencies between our findings and those from other domestic studies in China are likely due to differences in the two data sources, as the MCMS and DSPs data sets use different sampling frameworks and data collection methods [19]. Although both data sources adopted multistage cluster random sampling to select surveillance points, the stratification base differ and surveillance points greatly differ between the DSPs and the MCMS. Specifically, the DSPs used urbanization rate, population and mortality rate as stratification indicators while the MCMS relies on a range of social and economic indicators to classify potential surveillance points, including employment rate, age structure of the population, level of education, level of literacy, crude birth rate, crude death rate, infant death rate and gross domestic product (GDP) per capita [20]. Further, the 161 surveillance points of the DSPs are different from the 336 surveillance covered by the MCMS [6] and death data are reported by local hospitals and CDCs for the DSPs but by local maternal and child health institutions for the MCMS [20].

Our analysis using the DSPs estimates a suffocation mortality rate of 4.45 per 100 000 population of children ages 0-4, a rate that is extraordinarily lower than the only previously-published national study on this topic, using MCMS data, which reported 148.7-95.3 per 100 000 under-five children died of unintentional suffocation from 2000 to 2010. Our rate from the DSPs data set is comparable to GBD estimates over the same time period (8.42 to 5.33 per 100 000 persons) [1] as well as reports from Hunan province (15.82 to 7.74 per 100 000 persons from 2009 to 2014) [2], but lower than Sichuan province (238.0 to 69.8 per 100 000 persons from 2001 to 2013) [7].

The mortality disparities between the demographic groups we studied – higher suffocation rates among certain age (under-one vs 1- to 4-year-old children), sex (boys vs girls) and location (rural vs urban) groups generally concord with previous research in China [1,2,4-7] as well as other countries like Japan and Iran [17,21]. Our result that under-one children had a higher mortality rate from unintentional suffocation than older children also matches results from many other countries [17,18,22,23].

High injury rates in boys compared to girls have been reported in many countries [24]. The boy-girl gap in injury risks is hypothesized to result from differences in biological temperament, cognitive strategies, exposure opportunity and gender socialization [25]. Boys tend to take more risk, engage in more dangerous activities and behave more impulsively and fearlessly than girls. These behavior patterns may have biological or temperamental roots, but they also may be socialized by parents and other adults, as girls in many cultures receive more cautious care and guidance in potentially hazardous situations [25].

Urban-rural differences in child injury mortality in China can be interpreted as the results of three factors: inadequate adult supervision for left-behind children in rural areas [19], low safety awareness and less knowledge among rural adult caretakers of children [25], and relatively weak prehospital aid and hospital treatment for the injured children [8]. There may also be exposure factors, with children living in rural areas exposed to greater safety risks than children in urban settings.

Our results concerning the epidemiology of the mechanism of unintentional suffocation deaths among Chinese children under age five are novel to the published literature. The findings that under-one children have higher risk of bed-related suffocation while 1- to 4-year-olds have higher risk of inhalation suffocation are in line with behavioral and physical development of children [15,26,27] but contradict results from some countries that under-one children were more likely to die from inhalation suffocation rather than bed-related suffocation [17,23]. In China, many parents and adult caretakers prefer to sleep with their children, especially when they are infants, in a single bed. They also tend to cover babies with thick quilts on cold days. They also greatly increase the risk of unintentional suffocation among infants and young children [2]. The pattern we detected in China also reflects the lack of injury prevention education for safe sleeping practices in China compared to many developed countries where systematic efforts have not been made to encourage safe sleep habits and reduce infant and young child suffocation injuries [28-31].

Our findings underline the urgency and importance of preventing unintentional suffocation among under-five children in China. Applying DSP-based mortality estimates, over 3700 children under the age of 5 died in 2016, equal to over 10 deaths per day. These deaths are largely preventable, and our results indicate the situation is becoming worse rather than better over time. Multiple factors have led to neglect of unintentional suffocation prevention efforts in China [30], including national laws and regulations which fail to assign responsibility of implementing interventions to a particular government agency [31]. Prevention strategies that have proven effective in other nations have been recommended but inadequately implemented through the China National Program for Child Development (2010-2020), released by the State Council of China [32]. If those prevention strategies were implemented and enforced strictly, they could greatly reduce the burden of unintentional suffocation injury among under-five children across China.

This study was limited by a few factors. First, the data quality of DSPs depends on many quality control measures (including surveys every three years in all surveillance points) [12], but efforts to correct underreporting and misclassification only every three years may hide potential mortality variations that occur more frequently. Innovative approaches should be developed to collect validation data annually, thus overcoming this limitation of the DSPs data. Second, because information concerning many valuable exposure variables for child unintentional suffocation are not collected by the DSPs (eg, place, time, activities, product information (if involved) related to fatal suffocation), we cannot conduct detailed analysis to interpret recent mortality changes with confidence. Last, the introduction of web-based reporting to DSPs in 2008 was reported to have caused mortality fluctuations around that year, including for other injury causes, [9] but we cannot separate such impact from the effect of other factors to explain the change of mortality over the study time period.

In conclusion, we report a nearly stable trend in unintentional suffocation mortality from 2006 to 2016 among children aged under-five years old, but a significant rise for children aged 1-4 years, especially for boys and rural children in that age group. Throughout the time period studied, boys and children living in rural areas were at higher risk of unintentional suffocation mortality than girls and children living in urban areas. Targeted prevention strategies should be taken to reduce unwanted deaths from unintentional suffocation, and especially to curb the increasing trend among 1- to 4-year age group. Priority might be given to the prevention of strangulation and suffocation in bed for children under age 1 and inhalation suffocation prevention for 1- to 4-year olds, as these were the leading causes of suffocation mortality among the two age groups we studied.
